# A Novel Pulmonary Valve Replacement Surgery Strategy Using Contracting Band for Patients With Repaired Tetralogy of Fallot: An MRI-Based Multipatient Modeling Study

**DOI:** 10.3389/fbioe.2021.638934

**Published:** 2021-05-19

**Authors:** Han Yu, Pedro J. del Nido, Tal Geva, Chun Yang, Zheyang Wu, Rahul H. Rathod, Xueying Huang, Kristen L. Billiar, Dalin Tang

**Affiliations:** ^1^School of Biological Science and Medical Engineering, Southeast University, Nanjing, China; ^2^Department of Cardiac Surgery, Boston Children’s Hospital, Boston, MA, United States; ^3^Department of Surgery, Harvard Medical School, Boston, MA, United States; ^4^Department of Cardiology, Boston Children’s Hospital, Boston, MA, United States; ^5^Department of Pediatrics, Harvard Medical School, Boston, MA, United States; ^6^Mathematical Sciences Department, Worcester Polytechnic Institute, Worcester, MA, United States; ^7^School of Mathematical Sciences, Xiamen University, Xiamen, China; ^8^Department of Biomedical Engineering, Worcester Polytechnic Institute, Worcester, MA, United States

**Keywords:** right ventricle, Tetralogy of Fallot, ventricle mechanical model, surgery simulation, active contraction band

## Abstract

Patients with repaired Tetralogy of Fallot (ToF), a congenital heart defect which includes a ventricular septal defect and severe right ventricular outflow obstruction, account for the majority of cases with late-onset right ventricle (RV) failure. Current surgery procedures, including pulmonary valve replacement (PVR) with right ventricle remodeling, yield mixed results. PVR with active band insertion was hypothesized to be of clinical usage on improving RV function measured by ejection fraction (EF). In lieu of risky open-heart surgeries and experiments on animal and human, computational biomechanical models were adapted to study the impact of PVR with five band insertion options. Cardiac magnetic resonance (CMR) images were acquired from seven TOF patients before PVR surgery for model construction. For each patient, five different surgery plans combined with passive and active contraction band with contraction ratio of 20, 15, and 10% were studied. Those five plans include three single-band plans with different band locations; one plan with two bands, and one plan with three bands. Including the seven no-band models, 147 computational bi-ventricle models were constructed to simulate RV cardiac functions and identify optimal band plans. Patient variations with different band plans were investigated. Surgery plan with three active contraction bands and band active contraction ratio of 20% had the best performance on improving RV function. The mean ± SD RV ejection fraction value from the seven patients was 42.90 ± 5.68%, presenting a 4.19% absolute improvement or a 10.82% relative improvement, when compared with the baseline models (38.71 ± 5.73%, *p* = 0.016). The EF improvements from the seven patients varied from 2.87 to 6.01%. Surgical procedures using active contraction bands have great potential to improve RV function measured by ejection fraction for patients with repaired ToF. It is possible to have higher right ventricle ejection fraction improvement with more bands and higher band active contraction ratio. Our findings with computational models need to be further validated by animal experiments before clinical trial could become possible.

## Introduction

Tetralogy of Fallot (TOF) is a common congenital heart disease. The symptoms include ventricular septal defect, pulmonary valve stenosis, aorta overriding, and right ventricular hypertrophy. Corrective surgery allows TOF patients to extend their life expectancy, and some patients manage to survive into adulthood (Murphy et al., 1993). Many repaired TOF survivors are left with some residual symptoms including pulmonary regurgitation causing progressive RV dysfunction and dilation (Geva et al., 2004; Kim and Emily, 2016). Anagnostopoulos et al. (2007) hypothesized that pulmonary valve cusp augmentation with pericardium would decrease pulmonary insufluciency and improve the early outcome for transatrial–transpulmonary TOF repair requiring transannular patch. Their procedure had some success in reducing the incidence of clinically significant postoperative pulmonary insufluciency (Anagnostopoulos et al., 2007). For pulmonary valve (PV) sparing repair procedure which may be associated with residual pulmonary stenosis, Sen et al. (2016) reported that valve-sparing transannular (VSTAR) repair had better short-term and comparable midterm results and may be appropriate for TOF repair in patients with small PV. For repaired TOF patients with pulmonary regurgitation or unsuitable for pulmonary valve sparing procedure, current surgical approach [pulmonary valve replacement (PVR)] yield mixed results with some patients failing to recover their RV function (Geva et al., 2004, 2010; Kim and Emily, 2016). Recent advanced techniques include transcatheter strategies and using tissue-engineered valves (Motta et al., 2017; Jones and Qureshi, 2018). Del Nido and Geva et al. (2004) proposed a RV remodeling surgery by trimming scar tissue on RV wall and replacing the original patch with a smaller one during the PVR. In a clinic trial (NIH 5P50HL074734, Geva, and del Nido), 34 TOF cases were randomly assigned to PVR and RV remodeling surgery as experimental group and other 30 TOF cases underwent PVR alone as control group. Results showed insignificant statistical difference in RV EF variance after the surgery between the two groups (−2 ± 7% vs. −1 ± 7%; *p* = 0.38) (Geva et al., 2004). In search for innovative PVR surgical procedures to improve postsurgery RV cardiac functions, computational simulations for a PVR procedure with active contracting band insertions were performed using cardiac magnetic resonance (CMR) data from one TOF patient to investigate the effect of band material stiffness variations, band length, and active contraction ratios (Yang et al., 2013). The initial modeling results were promising (Tang et al., 2013; Yang et al., 2013).

Recent development in computational modeling made it possible for patient-specific ventricle models to be used for heart disease study and surgical optimizations. Peskin (1977, 1989) pioneered ventricle models with free moving boundaries and introduced early cardiac simulation models using immersed boundary method. McCulloch et al. (1992) developed a three-dimensional finite element method for large elastic deformations of ventricular myocardium, presenting the first practical opportunity to solve large-scale anatomically detailed models for cardiac stress analysis. Kerckhoffs et al. (2007) presented a novel method to couple the finite element cardiac mechanical model into a closed-loop lumped circulation models. Sacks and Chuong (1993) and Billiar and Sacks (2000) used biaxial mechanical test to acquire ventricle anisotropic material properties. Saber et al. (2001) and Axel (2002) proposed early magnetic resonance imaging (MRI)-based ventricle mechanical analysis. Nordsletten et al. (2011) developed a solid/fluid coupled left ventricle model to quantitate blood flow, pressure distributions, and mechanical energy loss caused by viscous dissipation.

Efforts were also made in moving ventricular computational models closer to clinical and surgical applications (Tang et al., 2008, 2013; Yang et al., 2013; Deng et al., 2018; Yu et al., 2019, 2020). Using CT-based mechanical fluid-solid interaction (FSI) ventricle model, Deng et al. (2018) studied systolic anterior motion of the mitral valve in hypertrophic obstructive cardiomyopathy. Our previous work included using bi-ventricular model to search surgical options, identify possible factors for post-PVR outcome prediction, estimate right ventricle myocardium stiffness, and study the impact of patch size, scar tissue removal, and RV remodeling on right ventricular function (Tang et al., 2008, 2013; Yang et al., 2013; Yu et al., 2019). Our pilot study using ventricle mechanical model of one TOF patient showed the surgery plan of inserting three bands with band active contraction ratio of 20% could improve RV ejection fraction from 37.38 to 41.58% (Yu et al., 2020).

We hypothesized that PVR with active contracting bands would improve RV cardiac function measured by ejection fraction (EF). RV EF was selected since it is easy to calculate and it is the single most commonly used measure for RV cardiac function in clinical practice. It is sufficient for the RV function assessment of our preliminary study. The normal RV EF range is 47–68% for a healthy male and 50–72% for a healthy female, respectively (Alfakih et al., 2010). In lieu of risky surgery with animals or TOF patients, patient-specific computational ventricle models based on CMR imaging data were used to quantify ventricle motion and evaluate RV ejection fraction before and after the band insert surgery. CMR data from seven repaired TOF patients were used to construct a total of 147 models combining five different band insertion options and four different contraction ratios. Results from these models were analyzed to seek optimized band insertion options with the best post-PVR outcome.

## Materials and Methods

### Data Acquisition

**FIGURE 1:**
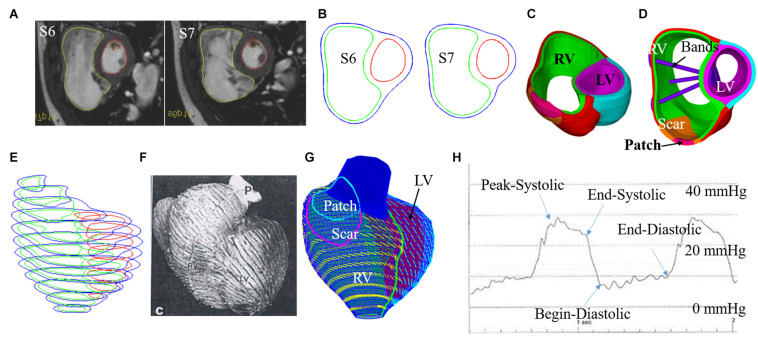
CMR-based model construction process and pressure conditions. **(A)** Selected CMR slices from a patient, end of systole. **(B)** Segmented contours. **(C)** Two-layer structure. **(D)**. Ventricle with three bands inserted. **(E)** Stacked contours. **(F)** RV fiber orientation from a TOF patient. **(G)** Model with fiber orientations. **(H)** Recorded RV pressure profile (Tang et al., 2008, 2013; Yang et al., 2013; Yu et al., 2019, 2020).

Boston Children’s Hospital Committee on Clinical Investigation approved this study. The approval number is IRB-CRM09-04-0237. Written informed consent was obtained from participants. CMR data was acquired from seven TOF patients 6 months before and after PVR (four males, average age: 31.81 years). Demographic and ejection fraction (EF) data of the seven TOF patients are given in Table 1. Post-PVR EF data served as a benchmark for us to check if the new surgery strategy with active contracting bands could provide better post-PVR cardiac outcome. CMR image segmentation was performed at Children’s Hospital-Boston, Harvard Medical School using a commercial software QMass (Medis Medical Imaging Systems, Leiden, the Netherlands). The locations of patch, scar, and valve were identified based on cine MRI, flow velocity data, and delayed enhancement CMR and with inspections by cardiac surgeon Dr. del Nido (PJdN, over 30 years of experience) who performed the PVR surgeries. Each acquired cardiac cycle data set included 30 discrete time points, and each time point had a 3D CMR image data. End-diastolic volume (EDV) and end-systolic volume (ESV) were computed with Simpson’s method. Figure 1 gives two sample CMR image slices at end ejection, their segmented contours, constructed 3D RV/LV model with scar, patch, and myocardium fiber orientation and recorded RV pressure *via* cardiac catheterization procedures.

**TABLE 1:** Patient demographic and CMR data.

Patient no.	Gender	Age (year)	Weight (kg)	Maximumpressure (mmHg)	RV EDV (ml)	RV ESV (ml)	RV EF (%)	PVR ΔEF (%)
P1	M	22.5	80	31.4	406.91	254.49	37.5%	1.49%
P2	M	47.7	95	31	408.76	254.79	37.7%	−2.56%
P3	M	43.5	123	65	665.06	464.05	30.2%	−15.22%
P4	F	38.5	49	28	328.79	195.97	40.4%	−3.35%
P5	M	11.6	38	36	204.17	121.26	40.6%	−8.41%
P6	F	14.3	58.5	29	204.00	104.30	48.8%	5.57%
P7	F	44.6	57.1	50	299.00	186.00	37.8%	−12.32%
Mean ± SD	–	31.81 ± 15.27	71.5 ± 29.7	38.63 ± 15.27	359.53 ± 158.61	225.84 ± 120.13	39.00 ± 5.53%	−4.97 ± 7.44%

*PVR, pulmonary valve replacement.*

*PVR ΔEF = post-PVR EF – pre-PVR RV.*

### Five PVR Surgical Plans With Active Contracting Bands and Band Active Contraction

**FIGURE 2:**
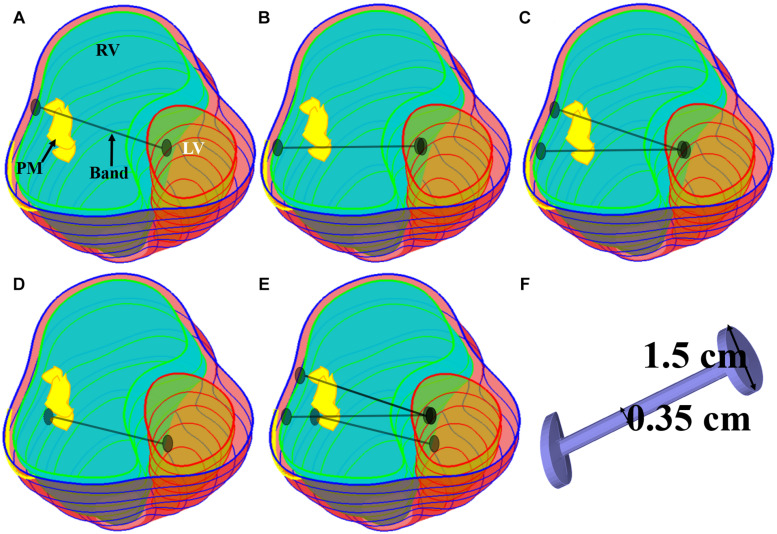
Five band insertion surgical plans. **(A)** Plan A: a band at anterior to the middle of papillary muscle (PM). **(B)** Plan B: a band at posterior to the middle of PM. **(C)** Plan C: double bands, plans A and B combined. **(D)** Plan D: a band at the base of the PM. **(E)** Plan E: combination of plans C and D. **(F)** Inserted band. PM, papillary muscle (Yu et al., 2020).

Five band insertion plans with band location and number variations are proposed to find optimal surgical plan using active contracting bands. An illustration of the five band plans is given by Figure 2. The five band plans included three plans with single band at different band locations, one plan with two bands, and one plan with three bands (Figure 2 and Table 2). The details were introduced in our previous publication (Yu et al., 2020). These five band plans led to 20 models for each patient with one passive band model and three active band models with different contraction ratios. Including the no-band baseline model, 21 models were constructed for each patient. Since we used seven TOF patients, a total of 147 models were included in our paper.

**TABLE 2:** Band model summary, band location and numbers, contraction ratios, and zero-load band length for all 21 RV/LV models.

Models	Plan	Band number	Contraction ratio	Zero-load band length (cm)	Band location
Baseline	–	0	–	–	–
A000	A	1	Passive	100%L	Anterior to the middle of PM
A010	A	1	10%	90%L	Anterior to the middle of PM
A015	A	1	15%	85%L	Anterior to the middle of PM
A020	A	1	20%	80%L	Anterior to the middle of PM
P000	B	1	Passive	100%L	Posterior to the middle of PM
P010	B	1	10%	90%L	Posterior to the middle of PM
P015	B	1	15%	85%L	Posterior to the middle of PM
P020	B	1	20%	80%L	Posterior to the middle of PM
AP000	C	2	Passive	100%L	Plan A and B combined
AP010	C	2	10%	90%L	Plan A and B combined
AP015	C	2	15%	85%L	Plan A and B combined
AP020	C	2	20%	80%L	Plan A and B combined
B000	D	1	Passive	100%L	The base of the PM
B010	D	1	10%	90%L	The base of the PM
B015	D	1	15%	85%L	The base of the PM
B020	D	1	20%	80%L	The base of the PM
APB000	E	3	Passive	100%L	Combination of plan C and D
APB010	E	3	10%	90%L	Combination of plan C and D
APB015	E	3	15%	85%L	Combination of plan C and D
APB020	E	3	20%	80%L	Combination of plan C and D

**Plan A models are models with a band at anterior to the middle of PM. They are named as AXXX, where A is short for anterior, and XXX represents band active contraction ratio, for example, 010 = 10%. Plan B models are models with a band at posterior to the middle of PM. They are named PXXX, where P is short for posterior. Plan D models has a band at the base of PM. They are called BXXX, where B is short for base. For multiband plans, plan C models are named as APXXX since plan C is the combination of A and B; and Plan E is named as APBXXX because plan E is the combination of A, P, and B. PM, papillary muscle.**

**FIGURE 3:**
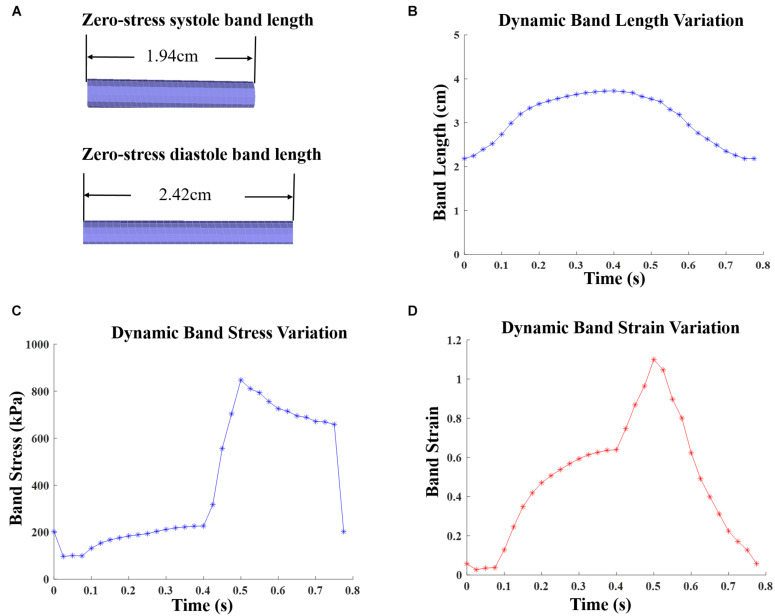
Zero-stress band length and dynamic band stress/strain variations for patient 5 surgery plan D model with 20% active contraction ratio in the entire cardiac cycle. **(A)** Zero-load band length at systole and diastole. **(B)** Dynamic band length variation. **(C)** Dynamic band stress variation. **(D)** Dynamic band strain variation.

The innovative PVR surgical plan using active contracting band was motivated by the fact that poor RV cardiac function indicated by low EF values were caused partially by RV’s weak contraction ability due to RV dilation. The contracting bands were used with the expectation that they would help RV to contract and improve its cardiac function. Passive elastic bands would not help since while they seem to be able to help RV to contract, they would hold the ventricle during its expansion (diastole) and defeat the purpose. Active contracting bands could help the ventricle to contract through active contraction and also allow the ventricle to re-expand through active relaxation. It is commonly known that myocardium active contraction is achieved by sarcomere shortening. However, active relaxation is equally important for the band models to work. Figure 3 gives plots of selected band zero-stress lengths in systole and diastole and band stress/strain curves in a cardiac cycle. When transitioning from diastole to systole (active contraction), the band zero-stress length changes from its diastole zero-stress length to systole zero-stress length (shortening) which results in strain and stress increases. When transitioning from systole to diastole (active relaxation), the band zero-stress length changed from its systole zero-stress length to diastole zero-stress length results in strain and stress decreases. Band material stress-strain curves are given in section “The RV/LV/Patch/Band Model and Material Models for Ventricle, Patch and Band.”

### The RV/LV/Patch/Band Model and Material Models for Ventricle, Patch, and Band

The RV/LV/patch/band model includes governing equations, boundary conditions, and material models for ventricle tissue, scar tissue, patch, and bands. The governing equations are the same for all structure components (Tang et al., 2008, 2013; Yang et al., 2013; Yu et al., 2019):

(1) $$\rho \,v_{i,t\,t}=\sigma_{i\,j,j},i,j=1,2,3;\mathrm{sum}\,\mathrm{over}\,j.$$

(2) $$\varepsilon_{i\,j}=\frac{1}{2}\,\left(v_{i,j}+v_{j,i}+v_{\alpha ,i}\,v_{\alpha ,j}\right),i,j,\alpha =1,2,3.$$

RV/LV combined geometry for each patient was obtained from CMR data and reconstructed following established procedures (Yang et al., 2013). Patch and scar locations were determined by our cardiac surgeon (PJ del Nido) and radiologist (T. Geva). No-slip conditions and natural boundary conditions were imposed automatically by ADINA (ADINA R&D, Watertown, MA, United States) at all interfaces of different structure components (ventricle, band, scar, and patch). RV and LV inner-pressure conditions were prescribed as (Tang et al., 2008, 2013; Yang et al., 2013; Yu et al., 2019, 2020):

(3) $${p|}_{R\,V}=p_{R\,V}\,\left(t\right),{p|}_{L\,V}=p_{\text{LV}}\,\left(t\right)$$

where *p*_*RV*_ and *p*_*LV*_ were blood pressure conditions specified on RV and LV inner surfaces. Pressure on the RV/LV out-surface was set to be zero. Patch, scar, and band materials were assumed to be hyper-elastic, isotropic, nearly incompressible, and homogeneous. The isotropic Mooney–Rivlin strain energy function is given by (Tang et al., 2008, 2013; Yang et al., 2013; Yu et al., 2019, 2020),

(4) $$W_{\text{iso}}=c_{1}\,\left(I_{1}-3\right)+c_{2}\,\left(I_{2}-3\right)+D_{1}\,\left[\exp\left(D_{2}\,\left(I_{1}-3\right)\right)-1\right]$$

where *c*_1_, *c*_2_, *D*_1_, and *D*_2_ are material constants and *I*_1_ and *I*_2_ are the first and second invariants of Cauchy–Green strain,

(5) $$I_{1}=\sum C_{i\,i},I_{2}=\frac{1}{2}\,\left(I_{1}^{2}-C_{i\,j}\,C_{i\,j}\right)$$

where *C*_*ij*_ is the Cauchy–Green deformation tensor. Ventricle tissue material was assumed to be hyper-elastic, anisotropic, nearly incompressible, and homogeneous. The non-linear anisotropic modified Mooney–Rivlin model was obtained by adding an additional anisotropic term in Eq. (4) (Yang et al., 2013):

(6) $$W=W_{\text{iso}}+W_{\text{aniso}}$$

(7) $$W_{\text{aniso}}=\frac{K_{1}}{K_{2}}\,\left(\exp\,{\left(I_{4}-1\right)}^{2}-1\right)$$

where *I*_4_ = *C*_*ij*_(*n*_*f*_)*_*i*_*(*n*_*f*_)*_*j*_*, *n*_*f*_ is the fiber direction, and *K*_1_ and *K*_2_ are material constants. With parameters chosen properly, the modified Mooney–Rivlin model described in Eq. (6) could fit the directly measured myocardium stress-strain data from our biaxial test experiment (Yang et al., 2013; Yu et al., 2019, 2020). In our models, patent-specific ventricle material parameter values were selected to match CMR-measured volume data.

A two-layer construction process was used to make our RV/LV models to take myocardium fiber orientations into consideration (see Figure 1D). Patient-specific fiber orientation data was not available for our study. Fiber orientation data from available literature were used in our models (Sanchez-Quintana et al., 1996; Nash and Hunter, 2000; Hunter et al., 2003). Fiber orientations were specified for every element on the inner and outer layers of our models. For left ventricle, the fiber orientation was approximately −60° (relative to circumferential direction) at the outer layer and +80° at the inner layer. RV fiber orientation was −45° at the outer layer and +40° at the inner layer (see Figure 1).

### Preshrink Process to Obtain Ventricle Zero-Load Geometries and Patient-Specific Ventricle Material Parameter Quantification

**FIGURE 4:**
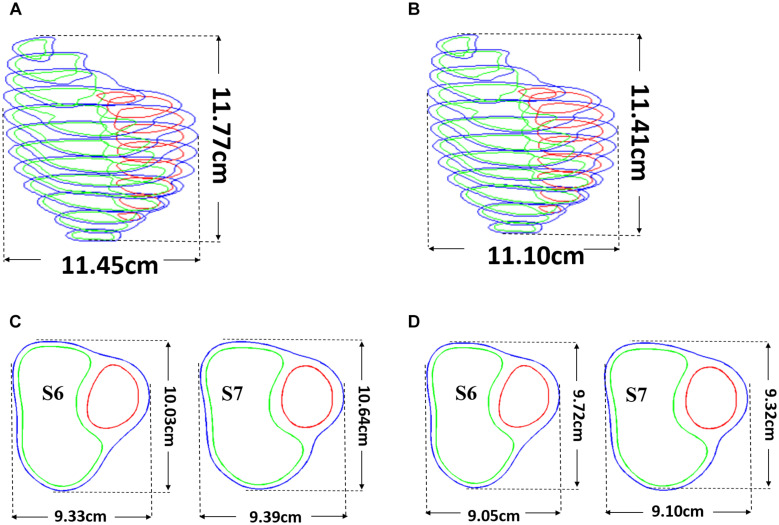
Pre-shrink process. **(A)** Stacked contours before pre-shrink. **(B)** Stacked contours after preshrink with a long-axis shrinking rate (3%). **(C)** Slices 6 and 7 before preshrink. **(D)** Slices 6 and 7 after preshrink, the shrink ratio for inner contours was 2–3%. The outer contours shrink ratio was determined to meet the conservation of mass of the total ventricular wall.

**FIGURE 5:**
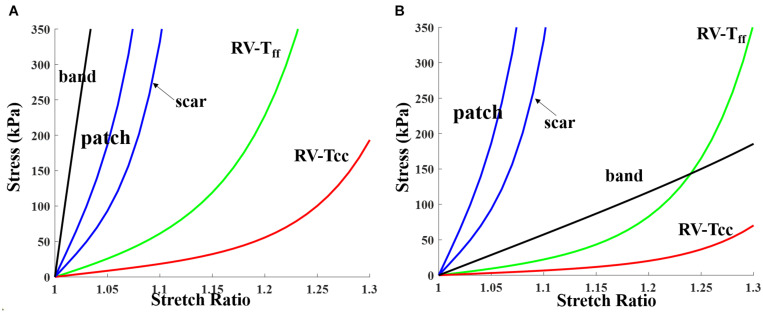
Stress-stretch curves for patch, scar, band, and myocardium tissue for RV inner layer (patient 5). Material parameter values are given in Table 3. **(A)** End systole. **(B)** End diastole. RV-T_*ff*_, RV stress in fiber direction; RV-T_*cc*_, RV stress in direction perpendicular to fiber direction.

Patient ventricle CMR images were obtained under *in vivo* conditions. Zero-load ventricular geometries were not available from *in vivo* CMR images and were obtained using a preshrink iterative process. In our modeling process, the approximate zero-load geometries were obtained by shrinking segmented contours on each slice (short-axis direction) with a short-axis shrinking rate and reducing distance between each slice (long-axis direction) with a long-axis shrinking rate (3%). The shrink ratio for inner contours was 2–3% based on the RV end-systole volume (minimum volume in a cardiac cycle) and the corresponding RV pressure. The outer contours shrink ratio was determined to meet the conservation of mass of the total ventricular wall. Figure 4 gives an illustration of the preshrink process. Three material parameter values *c*_1_, *d*_1_, and *K*_1_ in Eqs. (4)–(7) were adjusted iteratively until the relative error between the pressurized computational RV volume and CMR-measured *in vivo* volume data was less than 0.2%. This process was done semiautomatically using a secant method for fast convergence to *in vivo* RV volume. Figure 5 shows stress-stretch relations of patient 5 RV tissue, patch, scar, and band at begin filling and begin ejection, respectively. The corresponding material parameter values for those material models are shown in Table 3. For our seven no-band baseline models, the mean ± SD RV volume was 226.84 ± 121.20 ml at begin filling (same as end-systole volume ESV) and 359.19 ± 158.41 ml at begin ejection (same as end-diastole volume EDV), in agreement with the CMR data: 225.84 ± 120.13 ml at begin filling (*p* = 0.313) and 359.53 ± 158.61 ml at begin ejection (*p* = 0.438). Details were described in Tang et al. (2013, 2008), Yang et al. (2013), and Yu et al. (2019, 2020).

**TABLE 3:** Summary of parameter values of the Mooney–Rivlin models for patient 5 patch, scar, RV tissue, and band (*c*_2_ = 0 kPa).

Material/model	*c*_1_ (kPa)	*D*_1_ (kPa)	*D*_2_	*K*_1_ (kPa)	*K*_2_
Patch (isotropic)	26.52	26.52	9.0	0	–
Scar (isotropic)	13.26	13.26	9.0	0	–
Band (isotropic, end ejection)	900	0	–	0	–
Band (isotropic, end filling)	70	0	–	0	–
**Myocardium, end ejection**					
RV/LV inner layer (anisotropic)	7.64	2.41	3.0	36.55	3.0
RV/LV outer layer (anisotropic)	8.72	2.24	3.0	35.52	3.2
**Myocardium, end filling**					
RV/LV inner layer (anisotropic)	2.78	0.87	3.0	13.29	3.0
RV/LV outer layer (anisotropic)	3.17	0.82	3.0	12.92	3.2

### Solution Methods, Data Extraction, and Statistical Analysis

The 147 models were constructed and solved with ADINA using unstructured finite elements and the Newton–Raphson iteration method (Tang et al., 2008, 2013; Yang et al., 2013; Yu et al., 2019; Yu et al., 2020). Simulations were performed for several cardiac cycles until the solution relative differences between the last two cycles in *L*_2_ norm were less than 0.1%. The *L*_2_ norm of a given function *F* was computed with:

(8) $${\left\|F\right\|}_{L_{2}}=\sqrt{\int_{T_{k}}^{T_{k+1}}\left(\underset{V}{∭}F^{2}\text{d}v\right)\text{d}t,}$$

where *T* is the period (one cardiac cycle), *F* will be | *S*(*t*)−*S*(*t*+*T*)| as the function difference over one period where *S*(*t*) stands for either stress-*P*_1_ (maximum principal stress) or strain-*P*_1_ (maximum principal strain), the integration is taken over the entire RV domain and one cardiac cycle with discretization performed utilizing all available nodes and time steps. The results from the last period were recorded for analysis. For all 147 simulations, only three cycles were needed to obtain the solution. For each model, stress and strain data from 100 evenly spaced points for each slice of RV inner surface were extracted. Maximum principal stress and strain values were chosen to represent the stress and strain state of these points, and their mean values at begin filling (BF) and begin ejection (BE) were recorded as the stress and strain value of this model. RV Ejection fraction was used as the index (marker) for evaluating ventricle cardiac function. RV ejection fraction (EF) is defined as:

(9) $$\text{EF}=\frac{\mathrm{RV}\,\mathrm{EDV}-\mathrm{RV}\,\mathrm{ESV}}{\mathrm{RV}\,\mathrm{EDV}}\times 100\%$$

where RV EDV is right ventricle end-diastole volume and RV ESV is right ventricle end-systole volume. Higher EF value indicates that the ventricle is more efficient in pumping blood. Difference between pre- and post-PVR EF denoted by △EF was used to measure PVR (with and without active contracting bands) improvement:

(10) △⁢EF=post⁢-⁢PVR⁢EF-pre⁢-⁢PVR⁢EF.

Preoperation (no-band) RV/LV models were regarded as the baseline model. The paired test Wilcoxon signed rank test was used to compare the differences of EF, stress, and strain values between band and baseline models and the differences between different surgery plans. The test was performed using the function: *p* = signrank(a,b) from MATLAB statistical tool box.

## Results

### Three-Band Surgical Option Had Best Improvement for RV Ejection Fraction

Simulation results from the seven patients using five band options with band contraction ratio of 20% are given in Table 4. Surgery plan with three active bands (plan E) had the best RV EF improvement among the five band plans. The mean ± SD RV ejection fraction value from the seven patients with plan E was 42.90 ± 5.68%, representing a 4.19% absolute improvement or 10.82% relative improvement over the mean value of the baseline no-band models (38.71 ± 5.73%, *p* = 0.016). Absolute EF improvements for each patient varied from 2.87 to 6%. Mean ± SD ΔEF values of the seven patients for band options A–D were 2.51 ± 0.80%, 2.13 ± 0.53%, 3.36 ± 1.07%, 1.90 ± 0.40%, respectively. The two-band plan (option C) had the second best performance. Similar results were also found when the band active contraction ratio was 15 or 10%, respectively.

**TABLE 4:** RV ejection fraction (EF) and wall stress/strain data from models with band active contraction ratio of 20%.

Patients	Begin filling			Begin ejection			EF	ΔEF
		
	RV vol (ml)	Stress (kPa)	Strain	RV vol (ml)	Stress (kPa)	Strain		
**Plan A (1 band, option A)**								
P1	244.90	3.17	0.033	406.15	61.64	0.291	39.70%	2.32%
P2	248.20	3.20	0.029	408.42	46.61	0.314	39.23%	1.79%
P3	454.35	7.70	0.020	665.47	77.62	0.194	31.72%	2.12%
P4	186.00	3.46	0.019	327.59	54.74	0.308	43.22%	2.98%
P5	113.82	4.26	0.026	204.64	54.68	0.287	44.38%	4.11%
P6	100.39	3.13	0.022	204.14	58.03	0.418	50.82%	1.94%
P7	181.45	7.44	0.017	299.87	89.02	0.267	39.49%	2.41%
Mean ± SD	218.44 ± 118.7	4.62 ± 2.05	0.024 ± 0.006	359.47 ± 158.58	63.19 ± 14.85	0.297 ± 0.067	41.22 ± 5.85%	2.51 ± 0.80%
**Plan B (1 band, option B)**								
P1	246.47	3.16	0.032	407.07	64.72	0.285	39.45%	2.07%
P2	248.86	3.13	0.028	409.31	48.72	0.318	39.20%	1.76%
P3	455.69	6.99	0.019	662.07	76.83	0.192	31.17%	1.57%
P4	187.73	3.86	0.026	327.81	54.61	0.309	42.73%	2.49%
P5	115.45	4.52	0.030	203.80	54.03	0.287	43.35%	3.08%
P6	100.53	3.14	0.022	203.41	58.49	0.417	50.58%	1.70%
P7	181.57	7.30	0.018	299.74	88.49	0.266	39.43%	2.35%
Mean ± SD	219.47 ± 118.89	4.59 ± 1.82	0.025 ± 0.006	359.03 ± 157.84	63.70 ± 14.23	0.296 ± 0.067	40.84 ± 5.84%	2.13 ± 0.53%
**Plan C (2 bands, option C)**								
P1	240.64	3.21	0.033	405.83	63.98	0.289	40.70%	3.32%
P2	246.21	3.37	0.033	408.79	49.39	0.313	39.77%	2.33%
P3	447.69	8.10	0.022	664.29	79.06	0.199	32.61%	3.01%
P4	180.69	3.76	0.025	327.32	59.38	0.312	44.80%	4.56%
P5	111.12	3.31	0.025	203.22	51.38	0.286	45.32%	5.05%
P6	99.36	3.32	0.025	203.19	57.93	0.418	51.10%	2.22%
P7	179.26	7.91	0.020	299.65	89.08	0.266	40.18%	3.10%
Mean ± SD	214.99 ± 117.19	4.71 ± 2.26	0.026 ± 0.005	358.90 ± 158.61	64.32 ± 14.64	0.298 ± 0.066	42.07 ± 5.77%	3.36 ± 1.07%
**Plan D (1 band, option D)**								
P1	243.71	2.92	0.031	400.63	64.86	0.306	39.17%	1.79%
P2	248.88	2.88	0.026	407.35	46.87	0.310	38.90%	1.46%
P3	455.90	6.70	0.021	663.89	77.39	0.199	31.33%	1.73%
P4	189.25	4.33	0.026	327.44	58.42	0.305	42.20%	1.96%
P5	116.50	4.92	0.030	204.46	53.37	0.283	43.02%	2.75%
P6	100.97	3.24	0.024	204.56	56.94	0.419	50.64%	1.76%
P7	182.88	6.72	0.017	299.91	86.88	0.265	39.02%	1.94%
Mean ± SD	219.73 ± 118.5	4.53 ± 1.67	0.025 ± 0.005	358.32 ± 157.72	63.53 ± 14.08	0.298 ± 0.066	40.61 ± 5.81%	1.90 ± 0.40%
**Plan E (3 bands, option E)**								
P1	235.13	3.39	0.035	402.45	67.76	0.302	41.58%	4.20%
P2	243.04	3.53	0.037	408.20	51.56	0.315	40.46%	3.02%
P3	441.93	8.40	0.025	665.31	79.89	0.200	33.58%	3.98%
P4	178.64	4.11	0.028	327.18	59.69	0.312	45.40%	5.16%
P5	109.43	3.69	0.027	203.71	57.02	0.307	46.28%	6.01%
P6	98.20	3.73	0.030	203.52	59.39	0.423	51.75%	2.87%
P7	175.77	8.39	0.024	299.37	88.86	0.267	41.29%	4.21%
Mean ± SD	211.73 ± 115.66	5.03 ± 2.30	0.029 ± 0.005	358.53 ± 158.63	66.31 ± 13.48	0.304 ± 0.067	42.90 ± 5.68%	4.19 ± 1.11%

**ΔEF, difference between the current model EF and EF from baseline no-band model. Stress/strain values are mean values from all RV inner surface data points, 100 points per image slice.**

### Higher Band Contraction Ratio Had Better RV EF Improvement

Table 5 summarizes results from the seven patients using band option E (three-band model) with 0, 10, 15, and 20% band contraction ratios and their EF differences compared with the no-band baseline model. The models with 0% contraction ratio corresponded to models with passive bands. Mean ± SD ΔEF values of the seven patients for 10 and 15% band contraction ratio were 3.19 ± 1.00 and 2.27 ± 0.89%, respectively. Considering the results given in section “Three-Band Surgical Option had Best Improvement for RV Ejection Fraction”: mean ± SD ΔEF of seven patients for 20% band contraction ratio were 4.19 ± 1.11%, models with 20% band contraction ratio had higher ΔEF and EF values than that with 15 and 10% band contraction ratios. Higher band contraction ratio improved RV EF more.

**TABLE 5:** RV EF and wall stress/strain data of plan E models (3 bands) with 0, 10, 15, and 20% band contraction ratios.

Patients	Begin filling			Begin ejection			EF	ΔEF
		
	RV vol (ml)	Stress (kPa)	Strain	RV vol (ml)	Stress (kPa)	Strain		
**Passive bands**								
P1	248.83	2.82	0.028	375.78	54.60	0.278	33.78%	−3.60%
P2	254.60	2.51	0.019	394.09	42.87	0.310	35.40%	−2.04%
P3	466.38	6.09	0.017	644.83	76.66	0.197	27.67%	−1.93%
P4	191.92	3.44	0.018	307.01	49.67	0.295	37.49%	−2.75%
P5	118.11	3.77	0.025	180.59	41.78	0.266	34.60%	−5.67%
P6	103.77	2.53	0.015	190.01	50.33	0.407	45.39%	−3.49%
P7	187.09	5.87	0.012	282.35	77.11	0.253	33.74%	−3.34%
Mean ± SD	224.39 ± 121.35	3.86 ± 1.52	0.019 ± 0.006	339.24 ± 157.82	56.15 ± 14.84	0.287 ± 0.064	35.44 ± 5.32%	−3.27 ± 1.26%
**Band active contraction ratio: 10%**								
P1	242.37	3.01	0.030	402.81	67.33	0.301	39.83%	2.45%
P2	249.85	2.79	0.025	407.67	48.72	0.314	38.71%	1.27%
P3	455.54	6.57	0.018	665.33	78.99	0.200	31.53%	1.93%
P4	186.16	3.54	0.021	327.86	58.67	0.313	43.22%	2.98%
P5	114.05	3.60	0.025	203.88	55.88	0.306	44.06%	3.79%
P6	101.27	2.90	0.020	203.72	58.17	0.422	50.29%	1.41%
P7	182.07	6.32	0.015	299.44	87.98	0.267	39.20%	2.12%
Mean ± SD	218.76 ± 118.86	4.10 ± 1.63	0.022 ± 0.005	358.67 ± 158.54	65.11 ± 13.93	0.303 ± 0.066	40.98 ± 5.77%	2.27 ± 0.89%
**Band active contraction ratio: 15%**								
P1	238.86	3.18	0.032	402.66	67.47	0.301	40.68%	3.30%
P2	246.70	3.14	0.031	407.99	50.66	0.315	39.53%	2.09%
P3	449.11	7.37	0.021	665.58	79.25	0.200	32.52%	2.92%
P4	182.61	3.80	0.024	327.52	58.99	0.312	44.24%	4.00%
P5	111.80	3.61	0.025	203.81	56.41	0.306	45.15%	4.88%
P6	99.80	3.29	0.025	203.63	58.74	0.422	50.99%	2.11%
P7	179.07	7.23	0.019	299.54	88.72	0.267	40.22%	3.14%
Mean ± SD	215.42 ± 117.36	4.52 ± 1.92	0.025 ± 0.005	358.68 ± 158.66	65.75 ± 13.68	0.303 ± 0.066	41.90 ± 5.72%	3.19 ± 1.00%
**Band active contraction ratio: 20%**								
P1	235.13	3.39	0.035	402.45	67.76	0.302	41.58%	4.20%
P2	243.04	3.53	0.037	408.20	51.56	0.315	40.46%	3.02%
P3	441.93	8.40	0.025	665.31	79.89	0.200	33.58%	3.98%
P4	178.64	4.11	0.028	327.18	59.69	0.312	45.40%	5.16%
P5	109.43	3.69	0.027	203.71	57.02	0.307	46.28%	6.01%
P6	98.20	3.73	0.030	203.52	59.39	0.423	51.75%	2.87%
P7	175.77	8.39	0.024	299.37	88.86	0.267	41.29%	4.21%
Mean ± SD	211.73 ± 115.66	5.03 ± 2.30	0.029 ± 0.005	358.53 ± 158.63	66.31 ± 13.48	0.304 ± 0.067	42.90 ± 5.68%	4.19 ± 1.11%

**RV, right ventricle; EF, ejection fraction. ΔEF is the difference between the current model EF and EF from baseline no-band model.**

Intuitively, it might be reasonable to expect that a passive elastic band may be able to improve ejection fraction since elastic bands would help the ventricle to contract. Our simulations indicated that that was not the case. Mean ΔEF of models with surgery option E and passive bands was −3.27%, and ΔEF values ranged from −5.67 to −1.93%. For surgery option A–D, mean ± SD ΔEF were −1.37 ± 0.50, −1.04 ± 0.44, −2.32 ± 0.85, and −1.23 ± 0.79%, respectively. RV EF decreased after passive bands were inserted. The reason is actually simple: passive elastic bands would not “relax” by themselves after they contracted. The bands would actually hold the ventricle and became resistance for ventricle expansion. Mean ± SD volume values of surgery options A–D with passive bands at begin ejection were 349.76 ± 158.75, 352.08 ± 157.36, 344.56 ± 157.62, 350.83 ± 157.53, and 339.24 ± 157.82 ml, respectively, whereas the baseline model was 359.19 ± 158.41 ml. On the other hand, active bands help ventricle to contract by active contraction and would not resist ventricle expansion since active bands would “relax” through active relaxation (Table 6).

**TABLE 6:** Mean ± SD RV ejection fraction and wall stress/strain data of seven TOF patients from 140 RV/LV models with active or passive band(s) and seven models without band.

Plan	Begin filling			Begin ejection			EF	ΔEF
		
	RV vol (ml)	Stress (kPa)	Strain	RV vol (ml)	Stress (kPa)	Strain		
Baseline	226.84 ± 121.2	4.22 ± 1.63	0.022 ± 0.008	359.19 ± 158.41	62.89 ± 14.81	0.292 ± 0.062	38.71 |’ 5.73%	
A000	225.48 ± 121.6	4.05 ± 1.56	0.020 ± 0.006	349.76 ± 158.75	60.13 ± 14.82	0.291 ± 0.065	37.34 ± 5.56%	−1.370.50%
A010	222.19 ± 120.26	4.19 ± 1.69	0.021 ± 0.006	359.66 ± 158.55	63.35 ± 14.52	0.297 ± 0.067	40.21 ± 5.86%	1.500.63%
A015	220.41 ± 119.53	4.38 ± 1.84	0.022 ± 0.005	359.41 ± 158.49	63.41 ± 14.52	0.297 ± 0.067	40.67 ± 5.87%	1.960.75%
A020	218.44 ± 118.7	4.62 ± 2.05	0.024 ± 0.006	359.47 ± 158.58	63.19 ± 14.85	0.297 ± 0.067	41.22 ± 5.85%	2.510.80%
P000	226.01 ± 144.44	4.17 ± 1.58	0.021 ± 0.007	352.08 ± 157.36	61.01 ± 14.74	0.293 ± 0.065	37.67 ± 5.52%	−1.040.44%
P010	223.02 ± 120.21	4.17 ± 1.56	0.022 ± 0.006	359.43 ± 157.91	63.45 ± 14.26	0.297 ± 0.067	39.89 ± 5.83%	1.180.37%
P015	221.34 ± 119.58	4.34 ± 1.66	0.023 ± 0.006	359.12 ± 157.82	63.57 ± 14.23	0.296 ± 0.067	40.33 ± 5.84%	1.620.44%
P020	219.47 ± 118.89	4.59 ± 1.82	0.025 ± 0.006	359.03 ± 157.84	63.70 ± 14.23	0.296 ± 0.067	40.84 ± 5.84%	2.130.53%
AP000	225.02 ± 121.37	3.88 ± 1.59	0.019 ± 0.006	344.56 ± 157.62	57.99 ± 15.86	0.287 ± 0.064	36.39 ± 5.43%	−2.320.85%
AP010	220.54 ± 119.53	4.03 ± 1.73	0.021 ± 0.005	358.93 ± 158.60	63.49 ± 14.89	0.297 ± 0.066	40.52 ± 5.79%	1.810.77%
AP015	217.91 ± 118.43	4.32 ± 1.95	0.023 ± 0.005	359.10 ± 158.72	63.95 ± 14.70	0.298 ± 0.066	41.28 ± 5.77%	2.570.91%
AP020	214.99 ± 117.19	4.71 ± 2.26	0.026 ± 0.005	358.90 ± 158.61	64.32 ± 14.64	0.298 ± 0.066	42.07 ± 5.77%	3.361.07%
B000	225.72 ± 121.25	4.08 ± 1.58	0.021 ± 0.006	350.83 ± 157.53	59.57 ± 14.05	0.293 ± 0.066	37.48 ± 5.76%	−1.230.79%
B010	223.21 ± 120.01	4.14 ± 1.54	0.022 ± 0.006	358.94 ± 157.96	63.17 ± 14.05	0.298 ± 0.066	39.73 ± 5.82%	1.020.32%
B015	221.48 ± 119.34	4.30 ± 1.59	0.023 ± 0.005	358.76 ± 157.84	63.11 ± 14.10	0.297 ± 0.066	40.19 ± 5.8%	1.480.35%
B020	219.73 ± 118.5	4.53 ± 1.67	0.025 ± 0.005	358.32 ± 157.72	63.53 ± 14.08	0.298 ± 0.066	40.61 ± 5.81%	1.900.40%
APB000	224.39 ± 121.35	3.86 ± 1.52	0.019 ± 0.006	339.24 ± 157.82	56.15 ± 14.84	0.287 ± 0.064	35.44 ± 5.32%	−3.271.26%
APB010	218.76 ± 118.86	4.10 ± 1.63	0.022 ± 0.005	358.67 ± 158.54	65.11 ± 13.93	0.303 ± 0.066	40.98 ± 5.77%	2.270.89%
APB015	215.42 ± 117.36	4.52 ± 1.92	0.025 ± 0.005	358.68 ± 158.66	65.75 ± 13.68	0.303 ± 0.066	41.90 ± 5.72%	3.191.00%
APB020	211.73 ± 115.66	5.03 ± 2.30	0.029 ± 0.005	358.53 ± 158.63	66.31 ± 13.48	0.304 ± 0.067	42.90 ± 5.68%	4.191.11%

### Stress/Strain Patterns Were Complex in Right Ventricle

**FIGURE 6:**
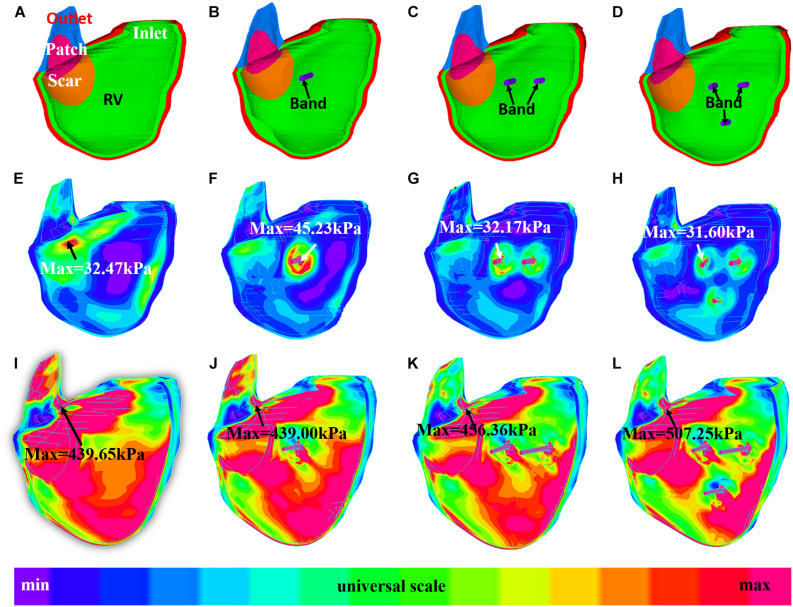
Stress plots of patient 5 from the no-band model and three active band models with band contraction ratio of 20%. **(A)** Model with no band, zero load. **(B)** Plan A: model with one band, zero load. **(C)** Plan C: model with two bands, zero-load. **(D)** Plan E: model with three bands, zero load. **(E)** Model with no band, begin filling. **(F)** Plan A: model with one band, begin filling. **(G)** Plan C: model with two bands, begin filling. **(H)** Plan E: model with three bands, begin filling. **(I)** Model with no band, begin ejection. **(J)** Plan A: model with one band, begin ejection. **(K)** Plan C: model with two bands, begin ejection. **(L)** Plan E: model with three bands, begin ejection.

**FIGURE 7:**
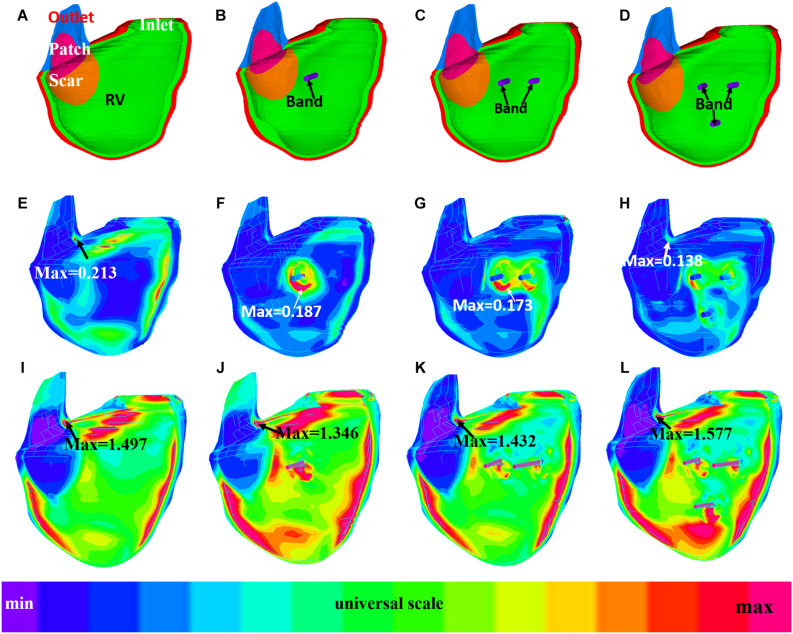
Strain plots of patient 5 from the no-band model and three active band models with band contraction ratio of 20%. **(A)** Model with no band, zero load. **(B)** Plan A: model with one band, zero load. **(C)** Plan C: model with two bands, zero load. **(D)** Plan E: model with three bands, zero-load. **(E)** Model with no band, begin filling. **(F)** Plan A: model with one band, begin filling. **(G)** Plan C: model with two bands, begin filling. **(H)** Plan E: model with three bands, begin filling. **(I)** Model with no band, begin ejection. **(J)** Plan A: model with one band, begin ejection. **(K)** Plan C: model with two bands, begin ejection. **(L)** Plan E: model with three bands, begin ejection.

Figures 6, 7 provides begin-ejection (maximum pressure) and begin-filling (minimum pressure) stress and strain plots on RV inner surface from four band models of patient 5. Stress and strain distributions have complex patterns. Band insertion changed local stress and strain distributions. Figures 6, 7 show that stress and strain values of RV tissues near band insert locations increased, whereas stress and strain relatively away from band decreased. Mean stress and strain values are given in Tables 2, 4, 5, showing variations due to band options, contraction ratios, and patient variations. There were very noticeable patient variations in stress/strain values. RV mean stress values from plan A varied from 46.61 kPa (P2) to 89.02 kPa (P7), a 91% increase. RV mean strain values from plan A varied from 0.194 (P3) to 0.418 (P6), a 115% increase. Patient mean stress/strain value variations for other band options were similar.

It should be noted that mean stress/strain values (average of seven patients) had very small variations for all five band options. At begin ejection, stress and strain values from models with active band were close to the baseline. RV mean ± SD stress values of the five plans with 20% band contraction ratio were 63.19 ± 14.85, 63.70 ± 14.23, 64.32 ± 14.64, 63.53 ± 14.08, and 66.31 ± 13.48 kPa, respectively. Meanwhile, baseline stress value was 62.89 ± 14.81 kPa. RV mean ± SD strain values of options A–E with 20% band contraction ratio were 0.297 ± 0.067, 0.296 ± 0.067, 0.298 ± 0.066, 0.298 ± 0.066, and 0.304 ± 0.067, which were also close to mean ± SD stress value of baseline models: 0.292 ± 0.062.

Table 5 comparing stress/strain values from models with different band contraction ratios. At begin ejection, average stress and strain values (seven patients) from models with 10% band contraction ratio were 16 and 5.6% higher than that from the passive band models. Average stress and strain values from models with 10, 15, and 20% band contraction ratios showed practically no differences (difference <2%).

## Discussion

### Motivation of the Innovative PVR With Active Contracting Band Insertion Procedures

How to manage the residual symptoms for repaired ToF cases such as pulmonary regurgitation has gradually become a great challenge for clinicians. Even though PVR could be an effective treatment for pulmonary regurgitation, other symptoms such as RV dysfunction may sometimes be irreversible after the PVR surgery (Geva et al., 2004, 2010; Kim and Emily, 2016). del Nido and Geva et al. (2010) proposed an aggressive surgical treatment including removing scar tissue and remodeling RV in order to improve RV function after PVR. However, the randomized clinical trial showed that the addition of surgical remodeling of the RV during PVR resulted in no measurable improvement in RV function (Geva et al., 2010).

PVR with active contracting band insertion aims to improve RV EF by assisting the ventricle to contract. In theory, the band should actively contract during systole, decreasing RV volume, and yet, the band should also be able to relax during diastole. These processes were reflected in our models: the mean ± SD RV volume values of models E020 at end ejection/begin filling were 211.73 ± 115.66 ml, which was lower than that of baseline models (226.84 ± 121.20 ml, *p* = 0.016). At end filling/begin ejection, no significant difference was found between E020 and baseline RV volume values (358.53 ± 158.63 ml vs. 359.19 ± 158.41 ml, *p* = 0.813) since the active bands were able to relax and did not resist the ventricle to expand in diastole phase (filling phase). Compared with our previous work on band insert surgery simulation, 147 models were constructed for seven patients with repaired TOF in this paper, vs. only one patient was used in previous paper (Yu et al., 2020). The purpose of this study was to demonstrate patient variations in post-PVR outcome using the proposed active contraction band options. Results from this seven-patient study were consistent with previous findings: RV ejection fraction values of all seven patients increased after the surgery with active contraction band insertion. The mean ± SD RV △EF value from the seven patients with plan E was 4.19 ± 1.11%, which is a significant improvement. Meanwhile, among all seven studied TOF patients, only two of them had increased EF values after pulmonary valve surgery (P1 and P6). The clinical mean ± SD ΔEF was -4.97 ± 7.44% based on actual patient pre- and post-PVR data. This is the proof of the concept that PVR with active contraction band insertion may have the potential to improve post-PVR RV cardiac function measured by ejection fraction for repaired ToF patients.

### Availability of Active Contracting Band

The idea of active band is completely theoretical currently. Our simulation provided the possible outcome of the surgery if such active contraction band could be made available. There are potential techniques that could be applied to manufacture active contracting band in the future including (a) using stem cells to cultivate muscle or (b) artificial mechanical muscle (Chou and Hannaford, 1996; Baar et al., 2005; Proulx et al., 2011; Sugimoto et al., 2017; Thomalla and Van deVen, 2018). Baar et al. (2005) used neonatal rat cardiomyogenic cells to construct a cylinder formed by cardiomyocytes which could be electrically induced to contract and showed positive inotropy and chronotropy (Baar et al., 2005). Proulx et al. (2011) described a band made by seeding human mesenchymal stem cells onto bundled fibrin microthreads and stitched through a collagen gel. Another viable solution is to use mechanical artificial active contraction device such as hydraulic or pneumatic McKibben actuator. McKibben actuator, also known as McKibben artificial muscle, is assembled with an internal elastic deformable tube surrounded by non-extensible threads weaved into an external mesh shell (Chou and Hannaford, 1996; Sugimoto et al., 2017; Thomalla and Van deVen, 2018). Such actuators were already widely used in robot technology. Takuma et al. designed a robot with McKibben actuators which could operate periodic motions including walking (Sugimoto et al., 2017). Maximum active contraction ratio of McKibben actuator could reach about 30% (Chou and Hannaford, 1996; Thomalla and Van deVen, 2018), which would be enough to meet the contracting requirement of our active contraction band used in PVR surgeries.

### Use of Ejection Fraction as a Measure of Surgical Outcome

RV EF was used as the measure of RV function in this paper because it is commonly used in practice and by many investigators, and it serves our demonstration purpose well. The concept is simple and calculation is easy. Outcome comparisons of different band options using a single indicator (EF) are straightforward and easy to understand. However, since it is only one number, it is lacking detailed local information for more careful evaluation of RV functions. 3D stress/strain distributions could compensate RV EV when detailed analysis is desired. RV kinetic energy due to its deformation in systole can be calculated as needed for a more detailed analysis. Since we currently have structure-only models, we would not be able to calculate the energy from the flow side and perform the analysis for that part. We are currently working on the corresponding fluid-structure interaction models. Full analysis from both structure and flow side will be reported when results become available. Those details will be helpful in band design and development of related tissue regeneration techniques (Tang et al., 2013).

It is true that EF is a crude measure of ventricular function (LV and RV), does not reliably reflect the functional status of the myocardium, and is sensitive to preload and afterload (so-called loading conditions). Despite these well-known limitations, EF has remained n inexplicably strong predictor of clinical outcomes in numerous diseases that affect the RV (e.g., repaired TOF) and the LV (e.g., aortic stenosis, hypertension, ischemic heart disease). These observation have been confirmed by numerous large studies. However, why EF is such a strong predictor of clinical outcomes is not fully understood. So, if the goal is to detect subtle abnormalities of heart muscle function, there are more sensitive tools. If the goal is to use it as a predictor of clinical outcomes (which is what ultimately matters), EF is an excellent marker.

### Validations

Validation is always ideal for computational modeling effort. It should be noted that the parameter determination process is a self-validation process in some sense. Our pre-PVR models were self-validated since patient-specific tissue material parameter values were carefully adjusted to match MRI-measured ventricle volume data. Since the ventricles remained to be the same post-PVR, using the same parameter values was natural for our post-PVR band models. Without actual clinical post-PVR data, using pre-PVR material properties gave us the best-effort approximations for our band models. Accuracies of simulation results including calculation of ventricle volumes and ejection fractions should be interpreted with that understanding.

Since materials with active-contraction properties are not currently available, direct validations using either patients or animal models are not possible at present time. Researchers have been working on myocardium regeneration for many years and encouraging progresses have been made. Clinical application of the active contraction band is what we would like to achieve in the future.

### Potential Clinical Applications

Possible potential clinical implementation of PVR with active contracting bands primarily depends on the availability of the availability of the active contracting bands. This modeling study is the proof of the concept that PVR with active contraction band insertion may improve post-PVR RV cardiac function measured by ejection fraction for repaired ToF patients. The mean ± SD RV △EF value from the seven patients with plan E was 4.19 ± 1.11% (all patients had improved EF), which is a significant improvement over the actual PVR surgery data ΔEF = −4.97 ± 7.44%. Meanwhile, among all seven studied TOF patients, only two of them had improved EF values after PVR (P1 and P6). A 9.16% average increase in RVEF compares favorably with published drug trials to treat heart failure where an improvement in LVEF of 3–4% resulted in a significant improvement in functional capacity (Aleksova et al., 2012).

### Limitations

This paper used structure-only models to save model construction manpower and computing time (147 3D models). Fluid-structure interaction did not include algorithm that allows a more realistic simulation. Clearly, FSI models will provide a more complete structural and flow information. However, for our purpose, EF was used to measure surgical outcome for the surgical strategies under investigation. The model parameters in both structure-only and FSI will be adjusted to match MRI-measured ventricle volume data. Therefore, structure-only or FSI models will give the same EF. Another reason that structure-only model could be used as a good approximation to calculate EF is that ventricle volume is mainly determined by the flow pressure acting on the ventricle inner surface. The flow stress activating on ventricle inner surface could be decomposed into pressure (normal component) and flow shear stress (shear component). The magnitude of flow shear stress is nearly negligible compared with pressure. That was the reason we used structure-only models to save time. FSI models will take far more time to construct and will be needed when we investigate flow behaviors, valve functions, ventricle remodeling, etc.

Lack of validation is definitely a limitation. This was discussed in section “Use of Ejection Fraction as a Measure of Surgical Outcome.”

Several other limitations exist in our modeling study: (a) patient-specific TOF RV/LV myofibril orientations should be included if available in the future; (b) localized tissue material properties were not available; and (c) flow behaviors and valve dynamics were not included in this study. We are working hard to obtain better ventricle data and improve our models.

## Data Availability Statement

The raw data supporting the conclusions of this article will be made available by the authors, without undue reservation.

## Ethics Statement

The studies involving human participants were reviewed and approved by the Boston Children’s Hospital Committee on Clinical Investigation. Written informed consent to participate in this study was provided by the participants’ legal guardian/next of kin.

## Author Contributions

TG, RR, and PN collected the CMR and blood pressure data. KB did the biaxial test. HY, CY, XH, ZW, and DT did model construction. HY did statistical analysis. HY and DT wrote and revised the manuscript. All authors contributed to the article and approved the submitted version.

## Conflict of Interest

The authors declare that the research was conducted in the absence of any commercial or financial relationships that could be construed as a potential conflict of interest. The reviewer AR-A declared a past collaboration with the authors TG and RR to the handling editor.

## References

[B1] Aleksova A. Masson S. Maggioni A. P. Lucci D. Urso R. Staszewsky L. (2012). Effects of candesartan on left ventricular function, aldosterone and bnp in chronic heart failure. *Cardiovasc. Drugs Ther.* 26 131–143. 10.1007/s10557-012-6370-8 22302146

[B2] Alfakih K. Plein S. Thiele H. Jones T. Ridgway J. P. Sivananthan M. U. (2010). Normal human left and right ventricular dimensions for MRI as assessed by turbo gradient echo and steady-state free precession imaging sequences. *J. Magn. Resonan. Imaging* 17 323–329. 10.1002/jmri.10262 12594722

[B3] Anagnostopoulos P. Azakie A. Natarajan S. Alphonso N. Brook M. M. Karl T. R. (2007). Pulmonary valve cusp augmentation with autologous pericardium may improve early outcome for tetralogy of Fallot. *J. Thorac. Cardiovasc. Surg.* 133 640–647. 10.1016/j.jtcvs.2006.10.039 17320558

[B4] Axel L. (2002). Biomechanical dynamics of the heart with MRI. *Annu. Rev. Biomed. Eng.* 4 321–347. 10.1146/annurev.bioeng.4.020702.153434 12117761

[B5] Baar K. Birla R. Boluyt M. O. Borschel G. H. Arruda E. M. Dennis R. G. (2005). Self-organization of rat cardiac cells into contractile 3-D cardiac tissue. *FASEB J.* 19 275–277. 15574489 10.1096/fj.04-2034fje

[B6] Billiar K. L. Sacks M. S. (2000). Biaxial mechanical properties of the natural and glutaraldehyde treated aortic valve cusp–Part I: experimental results. *J. Biomech. Eng.* 122 23–30. 10.1115/1.429624 10790826

[B7] Chou C. P. Hannaford B. (1996). Measurement and modeling of mckibben pneumatic artificial muscles. *IEEE Trans. Robot. Autom.* 12 90–102. 10.1109/70.481753

[B8] Deng L. Huang X. Yang C. Lyu B. Duan F. Tang D. (2018). Numerical simulation study on systolic anterior motion of the mitral valve in hypertrophic obstructive cardiomyopathy. *Int. J. Cardiol.* 266 167–173. 10.1016/j.ijcard.2018.01.062 29887442

[B9] Geva T. Gauvreau K. Powell A. J. Cecchin F. Rhodes J. Geva J. (2010). Randomized trial of pulmonary valve replacement with and without right ventricular remodeling surgery. *Circulation* 122 S201–S208. 20837914 10.1161/CIRCULATIONAHA.110.951178 PMC2943672

[B10] Geva T. Sandweiss B. M. Gauvreau K. Lock J. E. Powell A. J. (2004). Factors associated with impaired clinical status in long-term survivors of tetralogy of Fallot repair evaluated by magnetic resonance imaging. *J. Am. Coll. Cardiol.* 43 1068–1074. 10.1016/j.jacc.2003.10.045 15028368

[B11] Hunter P. J. Pullan A. J. Smaill B. H. (2003). Modeling total heart function. *Annu. Rev. Biomed. Eng.* 5 147–177. 10.1146/annurev.bioeng.5.040202.121537 14527312

[B12] Jones M. I. Qureshi S. A. (2018). Recent advances in transcatheter management of pulmonary regurgitation after surgical repair of tetralogy of Fallot. *F1000Res.* 7:679. 10.12688/f1000research.14301.1 29904583 PMC5981192

[B13] Kerckhoffs R. C. Neal M. L. Gu Q. Bassingthwaighte J. B. Omens J. H. McCulloch A. D. (2007). Coupling of a 3D finite element model of cardiac ventricular mechanics to lumped systems models of the systemic and pulmonic circulation. *Ann. Biomed. Eng.* 35 1–18. 10.1007/s10439-006-9212-7 17111210 PMC2872168

[B14] Kim Y. Y. Emily Rl (2016). Approach to residual pulmonary valve dysfunction in adults with repaired tetralogy of Fallot. *Heart* 102 1520–1526. 10.1136/heartjnl-2015-309067 27329296

[B15] McCulloch A. D. Waldman L. Rogers J. Guccione J. M. (1992). Large-scale finite element analysis of the beating heart. *Crit. Rev. Biomed. Eng.* 20 427–449. 1486784

[B16] Motta S. E. Lintas V. Fioretta E. S. Hoerstrup S. P. Emmert M. (2017). Off-the-shelf tissue engineered heart valves for in situ regeneration: current state, challenges and future directions. *Expert Rev. Med. Devices* 15 35–45. 10.1080/17434440.2018.1419865 29257706

[B17] Murphy J. G. Gersh B. J. Mair D. D. Fuster V. McGoon M. D. Kirklin J. W. (1993). Long-term outcome in patients undergoing surgical repair of tetralogy of Fallot. *N. Engl. J. Med.* 329 593–599. 10.1056/nejm199308263290901 7688102

[B18] Nash M. P. Hunter P. J. (2000). Computational mechanics of the heart, from tissue structure to ventricular function. *J. Elastic.* 61 113–141. 10.1007/0-306-48389-0_4

[B19] Nordsletten D. McCormick M. Kilner P. J. Hunter P. Kay D. Smith N. P. (2011). Fluid–solid coupling for the investigation of diastolic and systolic human left ventricular function. *Int. J. Numeric. Methods Biomed. Eng.* 27 1017–1039. 10.1002/cnm.1405

[B20] Peskin C. S. (1977). Numerical analysis of blood flow in the heart. *J. Com. Phys.* 25 220–252. 10.1016/0021-9991(77)90100-0

[B21] Peskin C. S. (1989). A three-dimensional computational method for blood flow in the heart. *J. Comp. Phys.* 81 372–405. 10.1016/0021-9991(89)90213-1

[B22] Proulx M. K. Carey S. P. Ditroia L. M. Jones C. M. Fakharzadeh M. Guyette J. P. (2011). Fibrin microthreads support mesenchymal stem cell growth while maintaining differentiation potential. *J. Biomed. Mater. Res. A.* 96 301–312. 10.1002/jbm.a.32978 21171149 PMC3058780

[B23] Saber N. R. Gosman A. D. Wood N. B. Kilner P. J. Charrier C. L. Firman D. N. (2001). Computational flow modeling of the left ventricle based on in vivo MRI data: initial experience. *Ann. Biomech. Eng.* 29 275–283. 10.1114/1.1359452 11339325

[B24] Sacks M. S. Chuong C. J. (1993). Biaxial mechanical properties of passive right ventricular free wall myocardium. *J. Biomech. Eng.* 115 202–205. 10.1115/1.2894122 8326727

[B25] Sanchez-Quintana D. Anderson R. H. Ho S. Y. (1996). Ventricular myoarchitecture in tetralogy of Fallot. *Heart* 17 280–286. 10.1136/hrt.76.3.280 8868990 PMC484521

[B26] Sen D. G. Najjar M. Yimaz B. Levasseur S. M. Kalessan B. Quaegebeur J. (2016). Aiming to preserve pulmonary valve function in tetralogy of fallot repair: comparing a new approach to traditional management. *Pediatr. Cardiol.* 37 818–825. 10.1007/s00246-016-1355-1 26921062

[B27] Sugimoto Y. Nakanishi D. Nakanishi M. Osuka K. (2017). Stability and joint stiffness analysis of legged robot’s periodic motion driven by McKibben pneumatic actuator. *Adv. Robot.* 31 441–452. 10.1080/01691864.2016.1273135

[B28] Tang D. Yang C. Geva T. del Nido P. J. (2008). Patient-specific MRI-based 3D FSI RV/LV/Patch models for pulmonary valve replacement surgery and patch optimization. *J. Biomech. Eng.* 130:041010. 10.1115/1.2913339 PMC2918812 18601452

[B29] Tang D. Yang C. Geva T. Rathod R. H. Yamauchi H. Gooty V. (2013). A multiphysics modeling approach to develop right ventricle pulmonary valve replacement surgical procedures with a contracting band to improve ventricle ejection fraction. *Comput. Struct.* 122 78–87. 10.1016/j.compstruc.2012.11.016 23667272 PMC3649854

[B30] Thomalla S. D. Van deVen J. D. (2018). Modeling and Implementation of the Mckibben actuator in hydraulic systems. *IEEE Trans. Robot.* 34 1593–1602.

[B31] Yang C. Tang D. Geva T. Rathod R. H. Yamauchi H. Gooty V. (2013). Using contracting band to improve right ventricle ejection fraction for patients with repaired tetralogy of fallot, a modeling study using patient-specific cmr-based two-layer anisotropic models of human right and left ventricles. *J. Thorac. Cardiovasc. Surg.* 145 285–293. 10.1016/j.jtcvs.2012.03.009 22487437 PMC3396738

[B32] Yu H. del Nido P. J. Geva T. Yang C. Wu Z. Rathod R. H. (2020). Multi-band surgery for repaired tetralogy of fallot patients with reduced right ventricle ejection fraction: a pilot study. *Front. Physiol.* 11:198. 10.3389/fphys.2020.00198 32265727 PMC7103653

[B33] Yu H. Tang D. Geva T. Yang C. Wu Z. Rathod R. H. (2019). Patient-specific in vivo right ventricle material parameter estimation for patients with tetralogy of fallot using mri-based models with different zero-load diastole and systole morphologies. *Int. J. Cardiol.* 276 93–99. 10.1016/j.ijcard.2018.09.030 30217422 PMC6324966

